# Adding Appropriate Fiber in Diet Increases Diversity and Metabolic Capacity of Distal Gut Microbiota Without Altering Fiber Digestibility and Growth Rate of Finishing Pig

**DOI:** 10.3389/fmicb.2020.00533

**Published:** 2020-04-09

**Authors:** Guang Pu, Pinghua Li, Taoran Du, Qing Niu, Lijuan Fan, Huan Wang, Hang Liu, Kaijun Li, Peipei Niu, Chengwu Wu, Wuduo Zhou, Ruihua Huang

**Affiliations:** ^1^Institute of Swine Science, Nanjing Agricultural University, Nanjing, China; ^2^Huaian Academy, Nanjing Agricultural University, Huaian, China; ^3^Industrial Technology System Integration Innovation Center of Jiangsu Modern Agriculture (PIG), Nanjing, China; ^4^Nanjing Agricultural University’s New Rural Research and Development Corporation of Huaian City, Huaian, China

**Keywords:** fiber intake, caecum, insoluble dietary fiber, 16S rRNA gene sequencing, VFA

## Abstract

The digestion ability of pigs to dietary fiber is derived from their intestinal microbiota, especially hindgut microbiota. However, tolerance of pigs to high dietary fiber and the changes of microbiota profile with fiber levels are still unclear. To investigate the changes of gut microbiota with dietary fiber and its relationship with fiber digestibility, we conducted comparative analyses of growth rate, apparent fiber digestibility, gut microbiota and volatile fatty acid (VFA) profiles in Chinese Suhuai pigs feeding diets with different defatted rice bran (DFRB) fiber levels. We found that dietary fiber level had no effect on the growth rate of Suhuai pigs. Although the apparent digestibility of Cellulose, insoluble dietary fiber (IDF) and total dietary fiber (TDF) decreased with dietary fiber level, we found that the apparent digestibility of Cellulose, IDF and TDF of Suhuai pigs was not changed when provided with diet containing 19.10% TDF (as feed basis). The pigs provided with diet containing 19.10% TDF had higher microbial richness, proportions of several fiber-degrading bacteria taxa at genus level and predicted microbial functions (such as carbohydrate metabolism, energy metabolism) in cecum compared to those fed with basal diet. In addition, the fiber-induced increasing of fiber-degrading bacteria promoted the VFAs metabolism, which potentially helped Suhuai pigs to maintain growth rate. However, as TDF reached to 24.11% (as feed basis), the apparent digestibility of fiber decreased and the positive effect on intestine microbiota in caecum were absent. Together, our data suggest that appropriate fiber level could increase the diversity and metabolic capacity of distal gut microbiota to improve the utilization efficiency of fiber resources without altering the growth rate of pigs.

## Introduction

Total dietary fiber (TDF) is the sum of a wide range of carbohydrates known as non-starch polysaccharides including pectins, cellulose, hemicellulose, β-glucans, and fructans as well as oligosaccharides and starch that are resistant to hydrolysis in the small intestine (Jarrett and Ashworth, 2018). According to its solubility, TDF can be divided into soluble dietary fiber (SDF) and insoluble dietary fiber (IDF) (Zhang et al., 2013). Although not fully digested, dietary fiber can change the nature of the contents of the gastrointestinal tract, which in turn affects the absorbtion of other nutrients and chemicals. And dietary fiber are the most commonly studied prebiotics (Gamage et al., 2018), which are generally fermented by the gut microbiota to yield energy and metabolic end products of microbial fermentation, such as volatile fatty acid (VFA) (Tremaroli and Backhed, 2012; Slavin, 2013; Janssen and Kersten, 2015). It is known that a diverse range of fiber-rich ingredients are added to pig diets all round the world. However, studies have long been reported that diets or ingredients with a high fiber content may negatively affect feed intake and nutrient digestibility in growing pigs (Kyriazakis and Emmans, 1995; Wilfart et al., 2007; Zhang et al., 2013). Despite these studies have indicated the great influence of dietary fiber to growth performance and nutrient digestibility, there is limited information available regarding the estimation of suitable dietary fiber inclusion level without affecting growth performance and nutrient digestibility.

Pigs lack the enzymes to degrade the bulk of dietary fibers. Therefore, these non-digestible carbohydrates were unaffected when pass through the upper gastrointestinal tract and are mainly fermented in large intestine by the anaerobic microbiota. Fermentation results in multiple groups of metabolites of which VFA are one of the primary metabolites (Greenberg et al., 2006). It was estimated that fiber fermentation products contribute 0.15 for growing-finishing pigs’ energy requirement (Dierick et al., 1989). Recently, Zhao et al. (2018) found that dietary corn bran or wheat bran may enhance the growth performance of weaned piglets via altering gut microbiota and improving butyrate production. However, there are few studies regarding the characteristics of caecal and colonic metabolites in finishing pigs when fed diets with different fiber levels.

Large scale study on the metagenomes of pigs revealed that 96% of the functional pathways found in the human gene catalog are present in the swine gut microbiome gene catalog, confirming the importance of pigs as human biomedical models (Xiao et al., 2016). The mammalian gastrointestinal tract harbors 500–1000 bacterial species that play important roles in the health and disease of the host (Guevarra et al., 2018; Birchenough et al., 2019). There are a variety of factors can modulate gut microbiota. Among them, age and diet are two of predominant modulator of the gut microbiota (Jeffery and O’Toole, 2013; Jones et al., 2019; Wang et al., 2019c). In addition, an overall increasing trend in microbial diversity and richness of the gut microbiome was observed during the pre-harvest lifespan of the pigs (Chen et al., 2017; Lu et al., 2018). The higher cellulolytic activity of adult pigs was also reviewed compared with young pigs (Lindberg, 2014). However, the concrete changes in the gut microbiota of finishing pigs to adapt to the increasing of dietary fiber level are not clear.

Chinese indigenous pig breeds had good performance in crude fiber digestion (Kemp et al., 1991; Fevrier et al., 2009). And Chinese Suhuai pig is a new national lean-type pig breed, containing 25% Chinese indigenous Huai pig ancestry and 75% Large White ancestry, possesses strong ability to digest high fibrous feedstuffs (Zhang et al., 2016; Wang et al., 2019a). White polished rice is the main food for more than 3 billion people in the world (García-Lara, 2010), and defatted rice bran (DFRB) is the byproduct of the rice milling process. DFRB is a potential fiber supplement for animal feeding. Previously, using DFRB as the main fiber source, we fed Chinese Suhuai pigs with diets containing 16.70%, 17.75%, 19.10%, 20.05%, and 24.11% TDF (as feed basis), respectively. We found that dietary fiber level had no effect on the average daily feed intake (ADFI), average daily gain (ADG) and F/G of Chinese Suhuai pigs, whereas the apparent digestibility of crude protein (CP) and ether extract (EE) decreased with dietary fiber level and the apparent digestibility of acid detergent fiber (ADF) decreased as TDF level was increased from 16.70% to 24.11%. However, the apparent digestibility of ADF had not changed as TDF level reached to 19.10% (Pu et al., 2019). We hypothesized that appropriate dietary fiber level potentially had a positive effect on the intestinal microbiota of Suhuai fattening pigs, which in turn enhanced the ability to digest fiber, and thus could potentially provide more VFAs as energy supplement for the growth of pigs.

Therefore, based on our previous experimental design and samples, the current study was carried out to further investigate the effects of diets with different fiber levels on growth rate, fiber digestibility, microbiota composition and its metabolic characteristics of Suhuai finishing pigs.

## Materials and Methods

### Animals and Treatment

Animals and experimental design of the current study is the same with our previous study (Pu et al., 2019). A total of 35 Suhuai barrows with body weight of 62.90 ± 0.78 kg were selected and allotted into 5 groups: the control group, and treatment I-IV using a completely randomized design. All pigs were fed by the Osborne Testing Stations System (OTSS, provided by OSB Livestock Technology Co., Ltd., Shanghai, China), which can accurately record daily intake, body weight individually. So, each pig is identified as a replicate and hence 7 replicates in each group. The whole process consisted of a 10-day pre-feeding period followed by a 28-day experimental period. There were five pens (2.5 m width × 5.25 m length), and pigs in the same group were housed in the same pen, respectively. All pigs were fed *ad libitum* by OTSS throughout the whole experiment period. The pigs had free access to water via a low-pressure bowl drinker. The temperature and relative humidity of the pig house were maintained at 24 ± 2°C and 69.82 ± 1.73% using direct-current frequency conversion floor heating air conditioner (Guangdong Eelaix Environmental Technology Co., Ltd., Guangdong, China). During pre-feeding period of 10 d, all pigs were fed with the basal diet. During the 28 days of the trial period, the pigs in the control group and treatment I–IV were provided with diets: the basal diet (the same basal diet used in pre-feeding period), 7%, 14%, 21%, and 28% of DFRB (as feed basis) substituted equivalent corn, respectively. The basal diet was formulated according to the Feeding Standard of Swine 60–90 kg Standard of Meat-fat Type Growing-finishing Pig (NY/T 65-2004). The diets were produced by Huaian Zhengchang Feed Co., Ltd. (Jiangsu, China). The chemical composition and analyzed nutritional contents of the experimental diets were shown in Table 1. The TDF content was 16.70%, 17.75%, 19.10%, 20.05%, and 24.11% (as feed basis), respectively. At the beginning of the experiment design, according to the “unique difference principle”, we not only used DFRB to replace corn to form fiber differences in each group, but also slightly adjusted the content of wheat bran, soybean meal and soybean oil in each group to make the calculated values of crude protein, amino acid and metabolic energy (ME) in each group’s feed formula close to the same. The analyzed chemical compositions of the DFRB and corn were shown in Supplementary Table S1. All animals were healthy and did not receive any antibiotic during the whole experimental period.

**TABLE 1:** Composition and analyzed nutrient levels of experimental diets (as-fed basis).

**Item**	**Groups**				
	**Control**	**Treatment**	**Treatment**	**Treatment**	**Treatment**
	**group**	**I**	**II**	**III**	**IV**
**Ingredients %**					
DM	88.56	88.68	88.93	89.16	88.46
Corn	68.61	62.00	55.00	48.00	41.00
Wheat bran	15.4	15.80	16.15	16.67	17.21
Defatted rice bran (DFRB)	0.00	7.00	14.00	21.00	28.00
Soybean meal	13.3	11.70	10.40	8.95	7.50
Soybean oil	0.00	0.84	1.83	2.78	3.74
Lysine	0.03	0.04	0.03	0.03	0.03
Salt	0.30	0.30	0.30	0.30	0.30
Limestone	0.82	0.85	0.85	0.85	0.85
CaHPO_4_	0.75	0.68	0.65	0.63	0.58
Choline	0.04	0.04	0.04	0.04	0.04
Premix^1^	0.40	0.40	0.40	0.40	0.40
**Nutrient level^2^**					
ME, MJ⋅kg^–1*^	12.13	12.13	12.22	12.27	12.31
CP %	15.60	16.67	16.13	15.73	16.40
EE %	5.19	5.08	5.32	5.27	5.38
NDF %	8.89	11.80	12.93	14.35	17.94
ADF %	5.53	6.25	6.53	7.08	8.13
IDF %	16.14	17.19	18.42	19.32	23.37
SDF %	0.52	0.56	0.68	0.73	0.82
TDF %	16.70	17.75	19.10	20.05	24.11
Cellulose %	4.06	4.43	4.71	5.09	5.79
Hemicellulose %	3.37	5.55	6.40	7.28	9.81
Lignin %	0.46	0.54	0.72	0.96	1.13
Calcium %	0.55	0.55	0.55	0.55	0.55
Available phosphorus %	0.27	0.27	0.27	0.27	0.27
L-lysine %	0.65	0.65	0.65	0.66	0.65
Methionine + cystine %	0.45	0.45	0.46	0.47	0.47

*^1^The premix was from Shanghai FuLangTe Animal Health Products Co., Ltd (Shanghai, China). The premix provided the following per kg of diets: vitamin A 8000 IU, vitamin D_3_ 1 500 IU, vitamin E 100 mg, vitamin K_3_ 4 mg, vitamin B_1_ 2 mg, vitamin B_2_ 8 mg, vitamin B_6_ 3 mg, vitamin B_12_ 0.04 mg, niacin 30 mg, pantothenic acid 35 mg, folic acid 0.6 mg, biotin 0.13 mg, Choline 150 mg, Fe 60 mg, Cu 5 mg, Zn 60 mg, Mn 10 mg, Se 0.15 mg and I 0.1 mg. ^2^DM, CP, EE, NDF, ADF, Hemicellulose, IDF, SDF, TDF, cellulose, and Lignin were measured values, while the other nutrient levels were calculated values.*

### Data Collection and Sampling

All pigs were slaughtered on the d 28 of experiment period. The data of growth performance were acquired from OTSS. The fresh fecal samples were collected at the end of the experiment period for determining nutritional apparent digestibility. The samples for determining nutritional apparent digestibility were mixed with 15 mL of 10% sulfuric acid solution and 200 g fresh fecal sample in plastic bag, then stored at −20°C. Meanwhile, caecal and colonic mucosa samples were collected for 16S rRNA gene amplicon sequencing. The samples of the caecal and colonic contents were collected for measuring the concentrations of VFAs and cellulase activity. The samples for conducting 16S rRNA gene sequencing, and measuring the concentrations of VFAs were immediately snap-frozen in liquid nitrogen and stored at −80°C.

### Growth Rate Analysis

Based on the real-time data of daily intake and body weight of each pig recorded by OTSS. The growth rate was obtained by linear regression of the body weight change of the experimental pigs. The slope of linear regression was considered as the growth rate.

### Nutritional Apparent Digestibility Analysis

The faeces and diet samples were dried at 65°C in the oven before determination. Acid-insoluble ash (AIA) was used as an endogenous marker to determine the nutritional apparent digestibility in diets. Diets, ingredients, and fecal samples were analyzed for dry matter (DM), AIA, acid detergent lignin, IDF, and SDF using standard procedures as previously described (Navarro et al., 2018). Total dietary fiber (TDF) in all samples were determined as the sum of IDF and SDF. The content of Hemicellulose and Cellulose were calculated as follows:

Hemicellulose = NDF − ADF

Cellulose = ADF − (Ash + Lignin)

Where: the data of neutral detergent fiber (NDF) and acid detergent fiber (ADF) were provided from our previous research (Pu et al., 2019).

The nutritional apparent digestibility was calculated as follow:

CAD(%)D=100×(1-(DC×FAIA)D/(DC×DAIA)F)

Where: CAD_D_ is the apparent digestibility of nutrients in experimental diets; DC_F_ is the nutrient content in feces; AIA_D_ is the acid insoluble ash in feeds; DC_D_ is the nutrient content in feeds; AIA_F_ is the acid insoluble ash content in feces.

The average intake of IDF, SDF and TDF were calculated as follows:

Average IDF intake = IDF content (%) × ADFI (g / d)

Average SDF intake = SDF content (%) × ADFI (g / d)

Average TDF intake = TDF content (%) × ADFI (g / d)

### VFAs Analysis

The VFAs concentrations in the content samples of caecum and colon were determined by gas chromatography (GC) as described previously (Lan et al., 2007). The sample peaks were identified by comparing their retention times with internal standards of acetate, propionate, butyrate, isobutyrate, valerate, isovalerate and metaphosphoric-crotonic acid.

### DNA Extraction and Sequencing Analysis

In this study, the apparent digestibility of Cellulose, IDF and TDF in treatment II was not different from that in the control group. While the apparent digestibility of Cellulose, IDF and TDF was significantly lower in treatment IV than that in the control group. We speculated that it may be the differences of distal gut microbiota, which caused the difference of fiber apparent digestibility. Therefore, 42 samples from control group, treatment II and IV (seven for each group) of caecum and colon were selected for 16S rRNA gene amplicon sequencing. Microbial DNA was extracted from caecal and colonic mucosal samples using the E.Z.N.A.^®^ soil DNA Kit (Omega Bio-tek, Norcross, GA, United States) according to manufacturer’s protocols.

The V3-V4 hypervariable regions of the bacteria 16S rRNA gene were amplified with primers 338F (5′-ACTCCTACGGGA GGCAGCAG-3′) and 806R (5′-GGACTACHVGGGTWTCTA AT-3′) by thermocycler PCR system (GeneAmp 9700, ABI, United States). Purified amplicons were pooled in equimolar and paired-end sequenced (2 × 300) was performed on an Illumina MiSeq platform (Illumina, San Diego, United States) according to the standard protocols by Majorbio Bio-Pharm Technology Co., Ltd. (Shanghai, China). Operational taxonomic units (OTUs) were clustered with 97% similarity cutoff using UPARSE (version 7.1^1^) with a novel ‘greedy’ algorithm performed chimera filtering and OTU clustering simultaneously. The taxonomy of each 16S rRNA gene sequence was analyzed by RDP Classifier algorithm^2^ against the Silva (SSU123) 16S rRNA database using confidence threshold of 70% (Wang et al., 2019b).

All the bacterial 16S rRNA amplicon sequencing data have been deposited in NCBI’s Short Read Archive under the accession number SRP239855.

### Activity of Fiber Degrading Enzyme Analysis

A 150 mL of 1% (w/v) solution of carboxymethyl cellulose, D-Salicin, microcrystalline cellulose and filter papers were used as the reaction substrate. A 50 μl crude enzyme solution extracted from each sample was added to determine the activity of carboxymethyl cellulose, microcrystalline cellulase, salicinase and filter paper enzyme using Microplate Reader at 600 nm (OD600), respectively.

### Statistical Methods

All data were presented as the mean ± SEM. Comparison of growth rate among treatment groups and control group by covariance analysis using SPSS 20.0. Contrast statements were used to determine the linear, quadratic effects of inclusion level of DFRB on the data of nutritional apparent digestibility and VFAs using SPSS 20.0. Comparing the differences of various indicators among groups were conducted using by one-way ANOVA followed by Least-significant difference (LSD) *post-hoc* test using SPSS 20.0. The correlations between the relative abundance of the bacterial genera, fiber apparent digestibility, average TDF intake and concentrations of VFAs were determined by Spearman’s correlation analyses (two-tailed test) using Origin 9.5. The phenotypic data of NDF and ADF came from our previous research (Pu et al., 2019). Statistical significance was defined as *P* < 0.05. Tendency was considered at 0.05 ≤ *P* < 0.10. Correlation network analysis among average fiber intake, microbiota and VFAs was conducted using Cytoscape 3.4.0 (Shannon et al., 2003).

## Results

### Growth Rate of Suhuai Pigs Was Not Adversely Affected by Dietary Fiber Level

**FIGURE 1:**
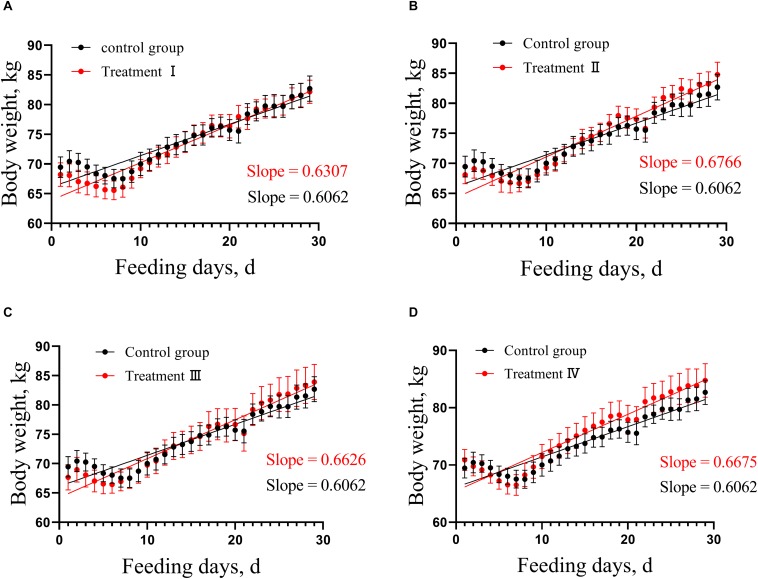
Comparison of linear growth rate between each of treatment groups and control. **(A)** Treatment I and control, **(B)** Treatment II and control. **(C)** Treatment III and control. **(D)** Treatment IV and control. According to the weight change of pigs in the treatment groups and the control, the linear regression was calculated, then the difference between the pair-wise linear regression was compared, respectively. Slope means linear growth rate.

In this study, analysis of covariance was used to compare the growth rate of treatment groups with control group, respectively. No difference was found in growth rate of pigs between each of the four treatment groups and the control group (Figure 1 and Supplementary Table S2).

### Appropriate Dietary Fiber Level Did Not Affect the Fiber Apparent Digestibility in Suhuai Pigs

**FIGURE 2:**
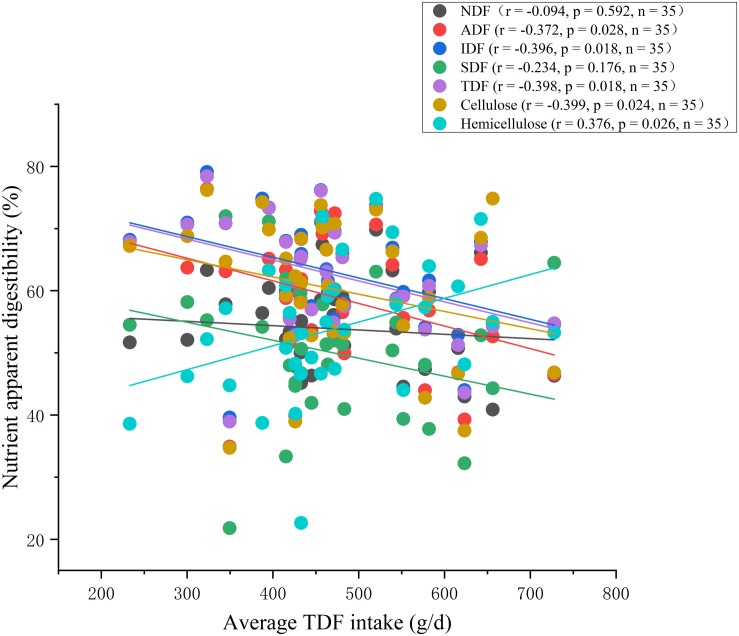
Correlation analysis between apparent digestibility of feed and average TDF intake.

At the end of the experiment period, apparent digestibility of Cellulose, IDF and TDF decreased linearly (*P* < 0.01), and changed quadratically with the increasing of dietary fiber level (*P* < 0.01) (Table 2). In addition, apparent digestibility of SDF had a decreased trend (linear, *P* < 0.10). While apparent digestibility of Hemicellulose increased linearly (*P* < 0.01), and changed quadratically (*P* < 0.01) (Table 2). Using LSD *post-hoc* test, we found that there was no difference in the apparent digestibility of Cellulose, IDF and TDF between treatment II and the control group, whereas apparent digestibility of Cellulose, IDF and TDF in treatment III and IV were significantly lower than that in control group (*P* < 0.05) (Table 2). Not surprisingly, significantly negative correlations were observed between feed apparent digestibility (e.g., ADF, Cellulose, IDF and TDF) and average TDF intake using Spearman’s correlation analysis (*P* < 0.05). While, significant positive correlation between average TDF intake and the apparent digestibility of Hemicellulose was observed (*P* < 0.05). In addition, the apparent digestibility of NDF and SDF had no correlation with average TDF intake (Figure 2).

**TABLE 2:** Responses of feed apparent digestibility to dietary fiber levels.

Items	**Groups**					**SEM**	***P* value**	
	**Control**	**I**	**II**	**III**	**IV**		**Linear^a^**	**Quadratic^b^**
Cellulose %	71.33	62.49	63.46	51.05*	52.52*	1.77	0.000	0.000
Hemicellulose %	41.78	51.95	61.51	55.17	58.95	1.83	0.002	0.001
IDF %	67.34	65.08	66.00	58.49*	57.42*	1.08	0.001	0.002
SDF %	57.16	53.45	54.09	40.12	55.02	1.99	0.056	0.145
TDF %	66.96	65.02	65.81	57.60*	56.79*	0.11	0.001	0.001

^*a*^*P* value for testing linear change with the increase of dietary fiber. ^*b*^*P* value for testing quadratic change with the increase of dietary fiber. *P < 0.05 treatment groups vs. control group. LSD *post-hoc* test was conducted.

### Suhuai Pigs Fed Diet With 19.10% TDF Had Higher Microbial Richness, and Proportion of Several Fiber-Degrading Bacteria Taxa at Genus Level

**FIGURE 3:**
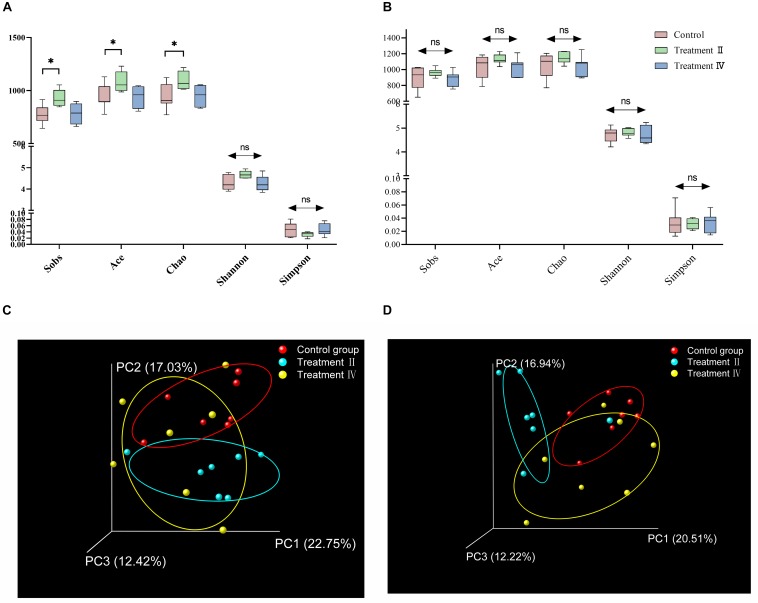
Diagram of microbial community’s comparison of between treatment groups and control group. Alpha diversity of caecal bacterial communities **(A)** and colonic bacterial community **(B).** Scatterplot from PCoA of caecal bacterial communities **(C)** based on unweighted UniFrac distance and colonic bacterial communities **(D)** based on Bray_Curtis distance. One-way ANOVA followed by LSD *post-hoc* test was conducted. *P < 0.05; ns, not significant.

A total of 1,214,947 and 1,481,284 high quality sequences were generated from caecal and colonic samples, with an average of 63,944 and 70,537 reads per caecal and colonic sample, respectively. In all cases, the established rarefaction curves showed that extra sampling would be of limited benefit (Supplementary Figure S1). Total OTUs obtained in caecum were as follows: at the level of OTUs, there were 1,376, 1,408, 1,339 in control group, treatment II and IV, respectively. And there were 96,132 and 58 special OTUs in the control group, treatment II and IV, respectively (Supplementary Figure S1E). Total OTUs obtained in colon were as follows: 1,458, 1,510 and 1,475 in control group, treatment II and IV, respectively. And there were 64, 97, and 91 special OTUs in the control group, treatment II and IV, respectively (Supplementary Figure S1F). Both the number of total OTUs and special OTUs of caecum and colon in treatment II were higher than those in the control group. One-way ANOVA analysis of alpha diversity revealed that the caecal microbial richness index (Sobs, Ace and Chao) of pigs in treatment II significantly higher than those in the control group (*P* < 0.05) (Figure 3A). No differences in diversity and richness of colonic microbiota of pigs between treatment groups and the control group were found (Figure 3B and Supplementary Table S3). Principal coordinate analysis (PCoA) based on unweighted UniFrac distance metric of the 16S rRNA sequences highlighted that there was a clear separation of the microbial community between treatment II and the control group (Adonis *P* < 0.05). Whereas no separation was observed between treatment II and the control group (Figure 3C). Dissimilarity in constitution of colonic microbiota of pigs between treatment II and the control group was noted using PCoA based on Bray_Curtis distance metric (Adonis *P* < 0.01), while no separation was observed between treatment II and control group (Figure 3D).

### Suhuai Pigs Fed Diet With 19.10% TDF Had Higher Proportions of Several Fiber-Degrading Bacteria Taxa at Genus Level

**FIGURE 4:**
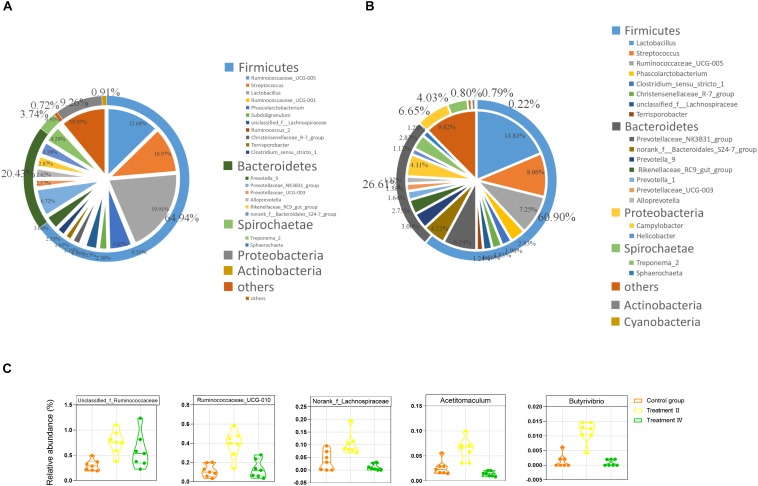
The gut microbiota composition of Suhuai pigs at phyla and genera level. **(A)** Relative distribution of the 20 most dominant bacterial genera and the phyla to which they belong in caecal samples. **(B)** Relative distribution of the 20 most dominant bacterial genera and the phyla to which they belong in colonic samples. Different shades of color represent different taxa. The pie chart depicts different genera. The outer ring around the pie chart depicts different phyla. Numbers in brackets denote the range of relative abundance of the bacteria. **(C)** Relative abundance of five fiber-degrading bacteria were significantly higher in treatment II compared to those in the control group (*P* < 0.01).

At the phylum level, Firmicutes, Bacteroidetes and Proteobacteria were 3 dominant phyla in two intestinal locations, which accounted for 65.92%, 20.32%, and 8.78% in caecum, 60.91%, 26.57%, and 6.68% in colon, respectively. At the genus level, *Lactobacillus* (14.44%), *Ruminococcaceae_UCG-005* (8.99%), *Streptococcus* (8.08%), *Prevotellaceae_NK3B31_group* (4.78%), *Phascolarctobacterium* (4.06%), *Campylobacter* (3.59%) and *Treponema_2* (3.01%) were the top seven genera in caecal samples (Figure 4A). While *Lactobacillus* (14.81%), *Streptococcus* (8.06%), *Ruminococcaceae_UCG-005* (7.25%), *Prevotellaceae_NK3B31_group* (6.24%), *norank_f__Bacteroidales_S24-7_group* (4.21%), *Campylobacter* (4.11%) and *Prevotella_9* (3.00%) were the top seven genera in colonic samples (Figure 4B). Two-tailed student’s *t* test was performed between treatment II, IV and the control group in the location of caecum and the results revealed that pigs in treatment II had significantly higher proportions of 35 taxa than those in the control group (*P* < 0.05, Supplementary Figure S2A). The top six flux changes in relative abundance were in the *Clostridium_sensu_stricto_1*, *Rikenellaceae_RC9_gut_group*, *Parabacteroides*, *unclassified_f_ Prevotellaceae*, *Ruminiclostridium_6* and *unclassified_f_ Ruminococcaceae.* Notably, comparing to those in the control group, treatment II had higher abundance of a constellation of fiber-degrading bacteria, particularly the *unclassified_f_Ruminococcaceae*, *Ruminococcaceae_UCG-010, norank_f__Lachnospiraceae* (Abdessamad et al., 2013), *Acetitomaculum* (Greening and Leedle, 1989) *Butyrivibrio* (Kalmokoff and Teather, 1997; Figure 4C) and the beneficial bacteria (e.g., *Erysipelotrichaceae_UCG-004* and *Akkermansia*, Supplementary Figure S2A) (*P* < 0.01). The proportions of 13 taxa were significantly different between treatment II and the control group. Of them, four genera of Ruminococcace had lower proportion in treatment II than those in the control group (Supplementary Figure SM1).

In the location of colon, pigs in treatment II group had significantly greater proportions of 22 taxa than those in control group. Of them, a reported bacterium (a genus of Lachnospiraceae) was related to fiber degrading (Abdessamad et al., 2013). While only 13 taxa had lower abundance in treatment II compared to those in the control group (*P* < 0.05) (Supplementary Figure S2C). The top six flux changes in relative abundance were in the *Campylobacter*, *Prevotella_9*, *Rikenellaceae_RC9_gut_group*, *unclassified_f_Lachnospiraceae*, *Helicobacter* and *Prevotella_1*. The proportions of 15 taxa were significantly different between treatment II and the control group (*P* < 0.05) (Supplementary Figure S2D).

### Dietary Fiber Promotes the Increase of the Functionality Activity of the Microbiota and Microbial Metabolites

**FIGURE 5:**
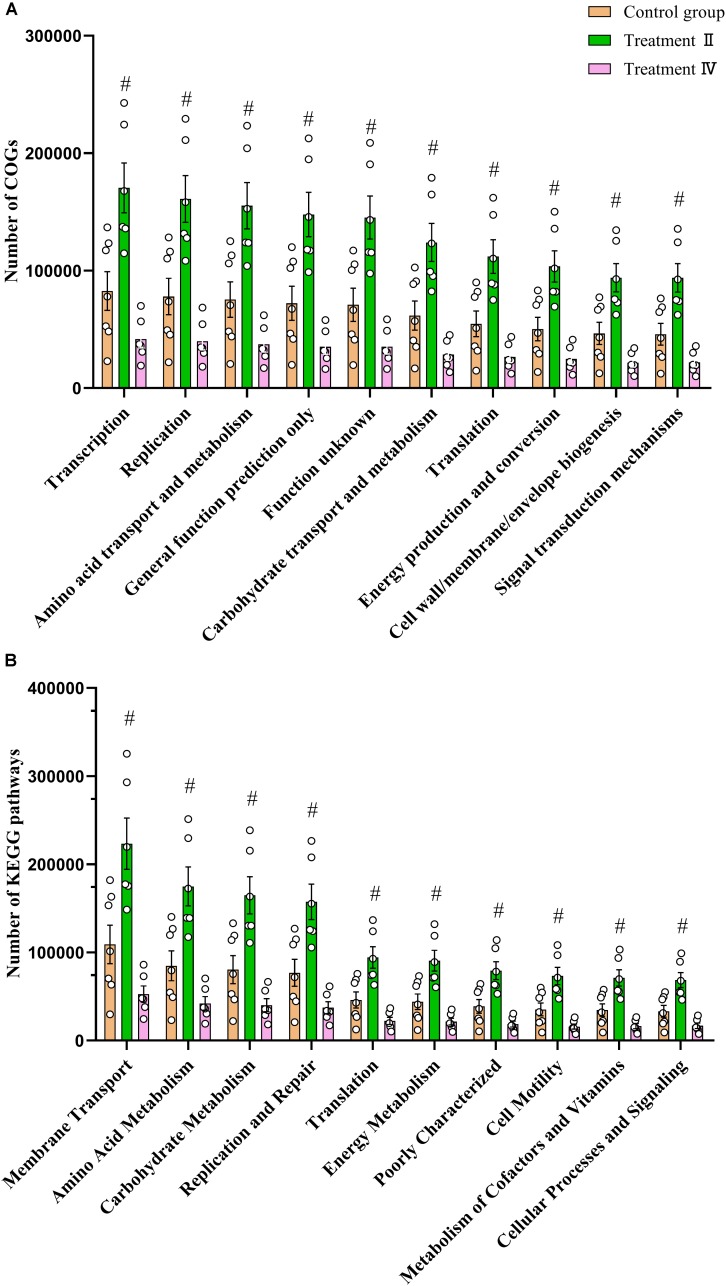
Comparison of caecal microbial functions between treatment groups and control group. **(A)** Comparison of the top 10 microbial functions of COG between treatment II, IV with control, respectively. **(B)** Comparison of the top 10 microbial functions of KEGG between treatment II, IV with control, respectively. One-way ANOVA followed by LSD *post-hoc* test was conducted. ^#^*P* < 0.01 between treatment II and control group.

We predicted microbial functions of different microbiota between treatment groups and the control group (*P* < 0.01) using Phylogenetic Investigation of Communities by Reconstruction of Unobserved States (PICRUSt) analysis referred to Cluster of Orthologous Groups of proteins (COG) database and Kyoto Encyclopedia of Genes and Genomes (KEGG) orthology database. Two-tailed student’s *t* test showed that all of the top 10 of microbial functions of COG and KEGG had higher abundance in treatment II compared to those in the control group (including Carbohydrate transport and metabolism, Amino acid transport and metabolism, Energy production and conversion and Metabolism of Cofactors and Vitamins) (*P* < 0.01) (Figures 5A,B). No different function was observed between treatment II and the control group. Similar results were found in the colon (Supplementary Figure S3).

In addition, we measured the activities of four enzymes involved in fiber degradation using ELISA assay kits. The results revealed that the salicinase activity in caecum of pigs in treatment II was significantly higher than that of those in the control group and treatment II (*P* < 0.05). While the microcrystalline cellulose activity in caecum of pigs in treatment II and IV were significantly lower than that of those in the control group (*P* < 0.01) (Supplementary Figure S4A). In colon, there was no significant difference in the activity of four fiber degrading enzymes between each of treatment groups and the control group (Supplementary Figure S4B).

We next determined the VFAs levels in caecum and colon using GC. The results showed the concentrations of acetate, propionate, isobutyrate and total VFA in caecum significantly increased with the dietary fiber level (Linear, *P* < 0.05) (Supplementary Table S4). And the concentrations of acetate and total VFA in colon significantly increased with dietary fiber level (Linear, *P* < 0.05). While the concentration of valerate significantly decreased (Linear, *P* < 0.05) (Supplementary Table S4). In addition, in the location of caecum, one-way ANOVA revealed that the concentrations of isobutyrate and isovalerate in treatment II were higher than those in the control group (*P* < 0.01). And the concentration of isobutyrate in treatment II were higher than that in the control group (*P* < 0.01) (Supplementary Table S4). In the location of colon, the concentration of acetate in treatment II was higher than that in the control group (*P* < 0.01) (Supplementary Table S4).

### Correlation Network Analysis Among Microbiota Composition, VFAs and Fiber Intake

**FIGURE 6:**
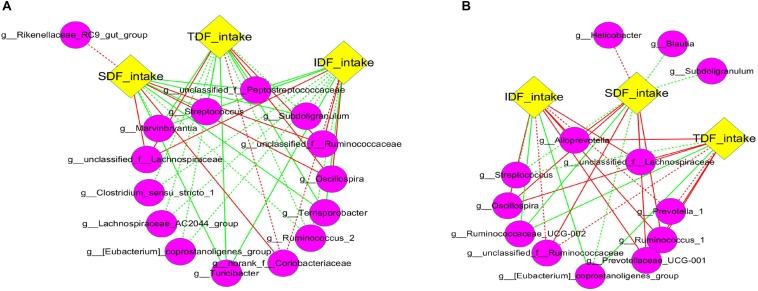
Correlation network analysis between fiber intake and microbiota. Networks display Spearman rank correlations between the relative abundance of bacteria at genus level and fiber intake in caecum **(A)** and colon **(B)**. The preceding letters “g” in the bracket represent genus bacteria. Solids represent the correlations with corrected values of 0.00 < *P* < 0.05, and dashed lines represent the correlations with corrected values of 0.05 ≤ *P* < 0.10. Red lines represent positive correlation and green lines represent negative correlation.

Although most fiber apparent digestibility decreased with the increase of DFRB substitution level, we noticed that fiber intake significantly increased (Supplementary Figure S5). We speculated that the increase of fiber intake may be one of critical reasons for the change of intestinal microbiota. Therefore, to investigate the specific effects of fiber intake on intestinal microbiota, we analyzed the correlation among the intake of three kinds of fibers (IDF, SDF, and TDF) and the main taxa (the top 50 of genera). Overall, different network structures were observed in each of the three types of fibers (Figure 6). Our results showed that *Oscillospira*, and some genera of Ruminococcace and Lachnospiraceae in caecum were significantly positively correlated with the intake of IDF, SDF and TDF (*P* < 0.05) (Figure 6A). Similar correlations were also found in colon (Figure 6B).

**FIGURE 7:**
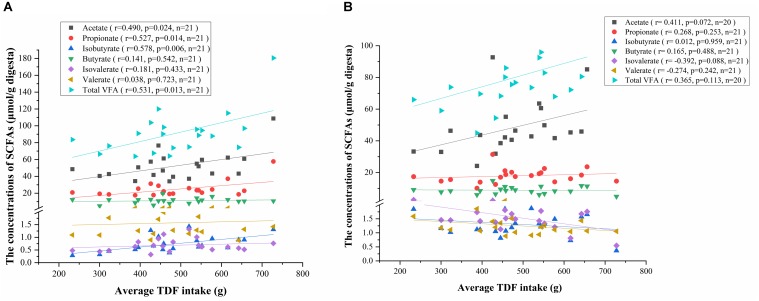
Correlation analysis between the concentrations of VFAs and average TDF intake. **(A)** Correlation analysis between the concentrations of VFAs in caecum and average TDF intake. **(B)** Correlation analysis between the concentrations of VFAs in colon and average TDF intake.

To investigate the relationship between VFAs production and fiber intake, we conducted a correlation analysis among the concentrations of VFAs and average TDF intake. Consistent with the results of one-way ANOVA, the concentrations of acetate, propionate, isobutyrate and total VFA in caecum positively correlated with the average TDF intake (Figure 7A), and the concentration of acetate in colon had a positive correlation trend with average TDF intake (*P* < 0.10) (Figure 7B).

**FIGURE 8:**
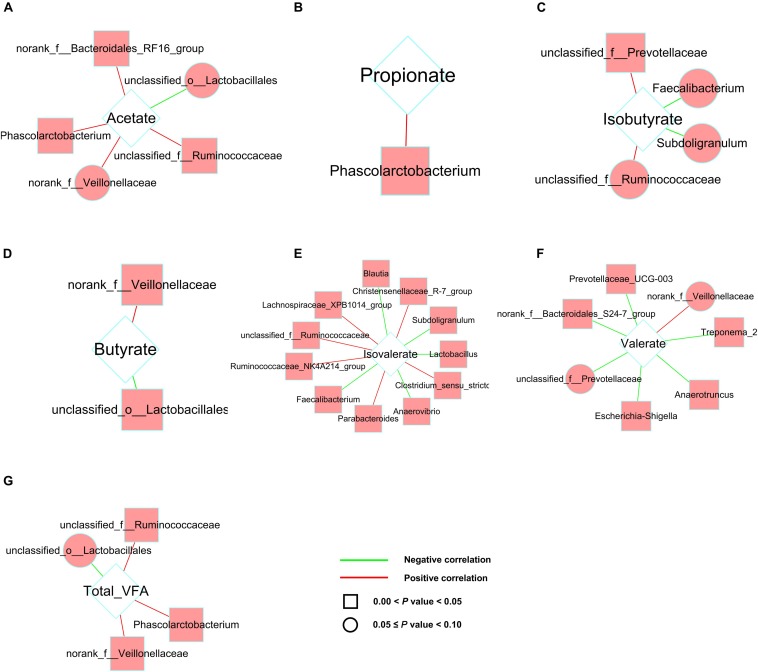
Correlation network analysis between the concentrations of VFAs and microbiota in caecum. Networks display Spearman rank correlations between the relative abundance of bacteria at genus level and the concentrations of acetate **(A)**, propionate **(B)**, isobutyrate **(C)**, butyrate **(D)**, isovalerate **(E)**, valerate **(F)**, and total VFA **(G)**. Square nodes represent the correlations with corrected values of 0.00 < *P* < 0.05, and round nodes represent the correlations with corrected values of 0.05 ≤ *P* < 0.10. Red lines represent positive correlation and green lines represent negative correlation.

In addition, to investigate how the gut microbial community was related to different patterns of VFAs productions, we analyzed the correlation between predominant taxa at genus level (the top 50 of genera) and VFAs productions (Figure 8). Correlation analysis revealed a number of significant correlations. In caecum and colon, the productions of VFAs were significantly correlated with various microbiota (*P* < 0.05). Of them, *Phascolarctobacterium* was positively correlated with acetic acid, propionic acid and total VFA production, and the genera of Ruminococcaceae were positively correlated with acetic acid and total VFA production in caecum (Figure 8). In colon, the genera of Peptostreptococcaceae and Ruminococcaceae were positively correlated with the production of acetic acid and butyric acid (Supplementary Figure S6).

## Discussion

### Growth Rate of Suhuai Pigs Was Not Adversely Affected by Dietary Fiber Level

With the further study on the physicochemical properties and nutritional functions of dietary fiber, Riva et al. (2019) found that dietary fiber is essential for the normal physiological metabolism of monogastric animals. Our results revealed that the linear growth rates of pigs in treatment I–IV had no difference compared to those in the control group, which indicated Suhuai fattening pigs could tolerate the diet with 28% DFRB. Similar results have been reported previously (Zervas and Zijlstra, 2002b,a). However, contradictory results have been reportedwell, they claimed indicating that the more soluble fibers increased the satiety via high water holding capacity, thus caused the decrease of feed intake (Kyriazakis and Emmans, 1995; Freire et al., 2000; García-Lara, 2010). The reasons that lead to different results with similar studies are complicated. Many factors can cause the different growth performance (e.g., genetic background, nutritional level, feeding management). We speculated that Suhuai pig breed, inheriting the rough feeding from Huai pig (a domestic pig breed), could adapt high fiber diet. Another possible hypothesis was that each diet had the same level of energy and protein, and met the energy needs of Suhuai pigs, which made pigs could have adequate energy to grow.

In addition, the calculated values of CP were 14.05, 14.02, 14.07, 14.07, and 14.08, respectively. However, the actual measured values of the CP showed in Table 1 had a little difference with the calculated value of CP. We regretted that the difference was exist between the calculated value and the actual measured value of CP, which may be due to the deviation between the calculated value and the measured value of each ingredient of formula. However, the differences of CP among these groups are smaller than the differences of fiber among these groups. Similarly, the difference of ME between the highest group and the lowest group is 0.18 MJ⋅kg^–1^, which less than that of fiber gradient. Therefore, we supposed that fiber was the main factor of no differences in body weight of pig. Of course, protein and ME might have little effect on body weight of pigs in each group.

### Adding Appropriate Fiber in Diet Increases the Diversity and Metabolic Capacity of Distal Gut Microbiota Without Altering Fiber Digestibility

Dietary fiber can be broadly categorized as insoluble or soluble, while the former resists fermentation, the latter is readily metabolized by gut bacteria (Singh et al., 2018). Similarly, some nutritional studies had shown that the digestibility of highly soluble ingredients such as soybean husk and beet meal were significantly higher than that of insoluble straw (Noblet and Goff, 2001). It was unexpected but need to be noted that in the present study, the apparent digestibility of SDF was much lower than that of IDF (no statistical test). Later, we found that most of the fibers contained in DFRB were insoluble, while the content of soluble fibers is very small (Supplementary Table S1). It has been reported when the content of insoluble fiber in diet was too high, the fiber structure would interfere with the contact opportunities of digestive enzymes and other nutrients (Bindelle et al., 2008). We speculated that the reason for the low digestibility of SDF might be due to that a very small amount of SDF had less chance of contacting with the corresponding degradation enzyme or the contact area was limited, therefore, it was not fully degraded. On the contrary, the content of IDF in DFRB was very high, the contact opportunity and contact area of IDF were more than SDF, thus it had higher digestibility. In addition, most studies compared the effects of soluble fiber diet and insoluble fiber diet on animal digestibility. However, the comparative study on the digestibility of SDF and IDF in the diet with insoluble fiber as the main diet is limited. Navarro et al. (2019) found that after feeding pigs with different content of Canola, total tract apparent digestibility of IDF was higher than SDF, which was consistent with our findings. In addition, most studies compared the effects of soluble fiber diet and insoluble fiber diet on animal digestibility. However, the comparative study on the digestibility of SDF and IDF in the diet with insoluble fiber as the main diet is limited.

Compared with insoluble substances, the addition of soluble fibrous substances had less influence on the absorption and utilization of other nutrients (Owusu-Asiedu et al., 2006; Renteria-Flores et al., 2008). The digestion of fibers in pigs mainly depends on the distal gut microbiota (Niu et al., 2019). Although fiber digestibility strikingly decreased with fiber intake in the present study, digestibility of cellulose, IDF and TDF of pigs could maintain when TDF level reached to 19.10%. To explain this phenomenon from the perspective of gut microbiota and clarify the characteristics of the distal gut microbiota and microbial metabolism, we determined the caecal and colonic microbiota of Suhuai pigs control group, treatment II and IV. The results revealed that the diet containing 19.10% TDF (treatment II) increased the richness of the microbiota and the proportions of several fiber-degrading bacteria taxa at genus level, such as two genera of Ruminococcaceae, one genus of Lachnospiraceae, *Acetitomaculum* and *Butyrivibrio*. Jackson MI et al. found adding fiber to food could increase the abundance of faecal Lachnospiraceae in dogs and improve intestinal health (Jackson and Jewell, 2019). Prebiotic functions of Lachnospiraceae were also discovered by Valcheva et al. (2019). Whereas few differences were seen between treatment IV (containing 24.11% TDF) and the control group. Wang et al. (2018) found that the richness of intestinal microbiota in the 15% alfalfa group was significantly lower than that in the control group. The authors explained that the results were supposed to be related to the abundance of some bacterial taxonomies, including phyla Proteobacteria, Actinobacteria, Gemmatimonadetes, and Chloroflexi in 15% alfalfa group were lower than that in the control group. In our present study, there were lots of genera had higher relative abundance in the diet with 19.10% TDF than those in the diet with 24.11% TDF (Supplementary Figure S2), which might be the immediate cause of the lower richness of microbiota in the diet with 24.11% TDF compared with those in the diet with 19.10% TDF. Singh et al. (2018); Jones et al. (2019) found too much dietary fiber could have an adverse effect on intestinal microbiota. Therefore, we speculated that the fiber level of the diet with 28% DFRB was too high, which might be not conducive to the growth of intestinal microbiota.

The microbial conversions of dietary fiber to monosaccharides in the gut involved a number of principal events (reactions) mediated by the enzymatic repertoire of specific members of the gut microbiota. Major end products from these fermentations are the VFAs (Koh et al., 2016). In this study, we found that Suhuai pigs fed the diet containing 19.10% TDF had higher abundance of microbial functions (e.g., carbohydrate metabolism, energy metabolism). In addition, the activity of salicinase in pigs fed the diet containing 19.10% TDF was significantly higher than those in the control group. Salicinase is a kind of fibrinolytic enzyme, can specifically act on β - cellobiose to produce glucose and hydrolyze a variety of β - glycoside bonds (Bayer et al., 1994; Kuhad et al., 2011). While pigs fed the diet containing 19.10% TDF had no advantage in activity of other three fiber-degrading enzymes. Therefore, we speculated that diet containing 19.10% TDF might promoted the decomposition of disaccharides into monosaccharides, thus promoted the metabolism of carbohydrate and energy and other microbial functions. Taken together, these findings might explain why the fiber digestibility of Suhuai pigs fed the diet containing 19.10% TDF did not decrease. However, the underlying mechanism is unknown and needs further study.

In addition, we found pigs provided with diet containing 19.10% had higher proportions of the genus *Akkermansia* and *Erysipelotrichaceae_UCG-004* than those in the control group (*Akkermansia muciniphilla* has been identified as a mucin-degrading bacteria that resides in the mucus layer and its abundance has been shown to positively correlate with enhancing intestinal barrier function and health profile of host (Karlsson et al., 2012; Everard et al., 2013; Clarke et al., 2014). And the members of family Erysipelotrichaceae may be the promising microbial targets for tackling metabolic disorders (Palm et al., 2014; Dinh et al., 2015).

### The Fiber-Degrading Bacteria Immensely Promote the Vfas Metabolism and Maintain the Growth Rate of Suhuai Pigs

VFAs, a series of bacterial fermentation end-products by degrading dietary fiber, provide pigs with 5–20% of their total energy (Ashida et al., 2011; Tilg and Kaser, 2011). Our previous study found that there was no difference in feed intake among groups, while the apparent digestibility of CP and EE decreased significantly with the increasing of dietary fiber level (Pu et al., 2019). Theoretically, the reduction of CP and ME digestibility would be detrimental to the growth performance of pigs. However, the growth performance was not affected in current study. In order to study the reason why apparent digestibility of CP, EE, and fiber decreased but the growth rate was not affected after the Suhuai pigs were fed with high-fiber diet. We measured the productions of VFAs in the caecum and colon. It was estimated that microbial fermentation products contribute 0.15 for growing-finishing pigs’ energy requirement (Dierick et al., 1989) and 0.3 for gestating sows’ energy requirement (Varel and Yen, 1997). In the present study, we found that concentrations of acetate, propionate, isobutyrate and total VFA in caecum and the concentrations of acetate and total VFA in colon increased with dietary fiber level. Moreover, we found that average TDF intake increased significantly with the fiber level. Furthermore, the correlation analysis revealed that the relative abundance of the genera of Ruminococcae, Lachnospiraceae and Peptostreptococcaceae increased significantly with the intake of SDF, IDF and TDF. Ruminococcae, Lachnospiraceae have been reported to be related to fiber degradation and VFAs productions (Abdessamad et al., 2013), and Peptostreptococcaceae has been reported to be related to low protein intake (Fan et al., 2017). Interestingly, these bacteria were concurrently positively correlated with the production of acetate, propionate, and total VFA in the cecum and colon. These results indicated that the increase of fiber intake promoted the metabolism of fiber-degrading related microbiota in pig hindgut and increased the productions of VFAs. Therefore, we speculated that, on one hand, the results of our study suggested that the increasing of VFAs productions might make up part of the energy needed for pig growth and development when the digestibility of CP and EE decreased. On the other hand, the growth performance was not affected, which might be because the experimental period of 28 days was relatively short and has not shown the adverse effect of high fiber diet on the growth performance of pigs.

In addition, it’s remarkable that, in the present study, pigs fed the diet with 24.11% TDF did not increase microbial diversity, fiber-degrading bacteria and the functionality activity of microbiota. However, the diets with 24.11% TDF increases the concentrations of VFAs in the cecum and colon of pigs. We thought there were two possible reasons may explain that: (1) our result revealed that dietary fiber levels promoted the fiber intake (Supplementary Figure S5), which promoted the total amount of fiber digestion; (2) the length of large intestine increased with the increase of dietary fiber level (linear, *P* = 0.083, unpublished) and we thus speculated that the diets with 24.11% TDF might increase the living space of distal gut microbiota and promote fiber digestion, thus promoted the increasing of VFAs concentrations.

Taken together, our findings suggest that appropriate high fiber level could increase the richness and metabolic capacity of distal gut microbiota without altering fiber digestibility and growth rate of finishing pigs.

## Conclusion

The increase of dietary fiber level did not affect the growth rate of Suhuai finishing pigs, but it promoted the increase of VFAs productions. When the TDF level reached to 19.10%, the apparent digestibility of fiber was not affected. Moreover, microbial alpha diversity and the abundance of five fiber-degrading bacteria was increased. However, as TDF reached to 24.11%, the apparent digestibility of fiber decreased and the positive effect on intestine microbiota in caecum were absent.

## Data Availability Statement

The raw data supporting the conclusions of this article will be made available by the authors, without undue reservation, to any qualified researcher.

## Ethics Statement

All experimental animals were performed according to Guidelines for the Care and Use of Laboratory Animals prepared by the Institutional Animal Welfare and Ethics Committee of Nanjing Agricultural University, Nanjing, China [Certification No: SYXK (Su) 2017-0007].

## Author Contributions

RH and PL designed research. GP, HW, LF, CW, HL, and KL collected and assembled the data. GP, RH, PL, TD, and QN performed analysis and interpreted data. RH, PN, and WZ provided financial support. GP wrote the manuscript. PL and RH contributed to the revision of the manuscript.

## Conflict of Interest

The authors declare that the research was conducted in the absence of any commercial or financial relationships that could be construed as a potential conflict of interest.

## Footnotes

1 http://drive5.com/uparse/

2 http://rdp.cme.msu.edu/

## Abbreviations

ADF: Acid detergent fiber
ADFI: Average daily feed intake
ADG: Average daily gain
AIA: Acid-insoluble ash
COG: Cluster of Orthologous Groups of proteins
CP: Crude protein
DFRB: Defatted rice bran
DM: Dry matter
EE: Ether extract
F/G: Feed to gain ratio
GC: Gas chromatography
IDF: Insoluble dietary fiber
KEGG: Kyoto Encyclopedia of Genes and Genomes
LSD: Least-significant difference
ME: metabolic energy
NDF: Neutral detergent fiber
OTSS: Osborne testing stations system
OUT: Operational taxonomic unit
PCoA: Principal coordinate analysis
PICRUSt: Phylogenetic Investigation of Communities by Reconstruction of Unobserved States
SRA: Sequence Read Archive
SDF: Soluble dietary fiber
TDF: Total dietary fiber
VFA: Volatile fatty acid.

## References

[B1] Abdessamad E. K. Fabrice A. Gordon J. I. Didier R. Bernard H. (2013). The abundance and variety of carbohydrate-active enzymes in the human gut microbiota. *Nat. Rev. Microbiol.* 11 497–504. 10.1038/nrmicro3050 23748339

[B2] Ashida H. Ogawa M. Kim M. Mimuro H. Sasakawa C. (2011). Bacteria and host interactions in the gut epithelial barrier. *Nat. Chem. Biol.* 8 36–45. 10.1038/nchembio.741 22173358

[B3] Bayer E. A. Morag E. Lamed R. (1994). The cellulosome–a treasure-trove for biotechnology. *Trends Biotechnol.* 12 379–386. 10.1016/0167-7799(94)90039-6 7765191

[B4] Bindelle J. Leterme P. Buldgen A. (2008). Nutritional and environmental consequences of dietary fibre in pig nutrition: a review. *Biotechnol. Agron. Soc.* 12 69–80.

[B5] Birchenough G. Schroeder B. O. Bäckhed F. Hansson G. C. (2019). Dietary destabilisation of the balance between the microbiota and the colonic mucus barrier. *Gut Microbes* 10 246–250. 10.1080/19490976.2018.1513765 30252606 PMC6546334

[B6] Chen L. Xu Y. Chen X. Fang C. Zhao L. Chen F. (2017). The maturing development of gut microbiota in commercial piglets during the weaning transition. *Front. Microbiol.* 8:1688. 10.3389/fmicb.2017.01688 28928724 PMC5591375

[B7] Clarke S. F. Murphy E. F. O’Sullivan O. Lucey A. J. Humphreys M. Hogan A. (2014). Exercise and associated dietary extremes impact on gut microbial diversity. *Gut* 63 1913–1920. 10.1136/gutjnl-2013-306541 25021423

[B8] Dierick N. A. Vervaeke I. J. Demeyer D. I. Decuypere J. A. (1989). Approach to the energetic importance of fibre digestion in pigs. I. Importance of fermentation in the overall energy supply. *Anim. Feed Sci. Tech.* 23 141–167. 10.1016/0377-8401(89)90095-3

[B9] Dinh D. M. Volpe G. E. Duffalo C. Bhalchandra S. Tai A. K. Kane A. V. (2015). The intestinal microbiota, microbial translocation and systemic inflammation in chronic HIV infection. *J. Infect. Dis.* 211 19–27. 10.1093/infdis/jiu409 25057045 PMC4326316

[B10] Everard A. Belzer C. Geurts L. Ouwerkerk J. P. Druart C. Bindels L. B. (2013). Cross-talk between *Akkermansia muciniphila* and intestinal epithelium controls diet-induced obesity. *Proc. Natl. Acad. Sci. U.S.A.* 110 9066–9071. 10.1073/pnas.1219451110 23671105 PMC3670398

[B11] Fan P. Liu P. Song P. Chen X. Ma X. (2017). Moderate dietary protein restriction alters the composition of gut microbiota and improves ileal barrier function in adult pig model. *Sci. Rep.* 7:43412. 10.1038/srep43412 28252026 PMC5333114

[B12] Fevrier C. Bourdon D. Aumaitre A. (2009). Effects of level of dietary fibre from wheat bran on digestibility of nutrients, digestive enzymes and performance in the European Large white and Chinese Mei Shan pig. *J. Anim. Physiol. Ann.* 68 60–72. 10.1111/j.1439-0396.1992.tb00618.x

[B13] Freire J. Guerreiro A. Cunha L. Aumaitre A. (2000). Effect of dietary fibre source on total tract digestibility, caecum volatile fatty acids and digestive transit time in the weaned piglet. *Anim. Feed Sci. Tech.* 87 71–83. 10.1016/S0377-8401(00)00183-8

[B14] Gamage H. K. A. H. Tetu S. G. Chong R. W. W. Bucio-Noble D. Rosewarne C. P. Kautto L. (2018). Fiber supplements derived from sugarcane stem, wheat dextrin and psyllium husk have differentin vitroeffects on the human gut microbiota. *Front. Microbiol.* 9:1618. 10.3389/fmicb.2018.01618 30072976 PMC6060387

[B15] García-Lara S. (2010). Cereal grains: properties, processing and nutritional attributes. *Crop Sci.* 50:2649 10.2135/cropsci2010.12.0005br

[B16] Greenberg N. A. Gassull M. A. Remy M. (2006). Short-chain fatty acids: ready for prime time? *Nutr. Clin. Pract.* 21 351–366. 10.1177/0115426506021004351 17119172

[B17] Greening R. C. Leedle J. A. Z. (1989). Enrichment and isolation of *Acetitomaculum ruminis*, gen. nov., sp. nov.: acetogenic bacteria from the bovine rumen. *Arch. Microbiol.* 151 399–406. 10.1007/bf00416597 2500921

[B18] Guevarra R. B. Hong S. H. Cho J. H. Kim B. R. Shin J. Lee J. H. (2018). The dynamics of the piglet gut microbiome during the weaning transition in association with health and nutrition. *J. Anim. Sci. Biotechnol.* 9:54. 10.1186/s40104-018-0269-6 30069307 PMC6065057

[B19] Jackson M. I. Jewell D. E. (2019). Balance of saccharolysis and proteolysis underpins improvements in stool quality induced by adding a fiber bundle containing bound polyphenols to either hydrolyzed meat or grain-rich foods. *Gut Microbes* 10 298–320. 10.1080/19490976.2018.1526580 30376392 PMC6546335

[B20] Janssen A. W. Kersten S. (2015). The role of the gut microbiota in metabolic health. *FASEB J.* 29 3111–3123. 10.1096/fj.14-269514 25921831

[B21] Jarrett S. Ashworth C. J. (2018). The role of dietary fibre in pig production, with a particular emphasis on reproduction. *J. Anim. Sci. Biotechnol.* 9:59. 10.1186/s40104-018-0270-0 30128149 PMC6091159

[B22] Jeffery I. B. O’Toole P. W. (2013). Diet-microbiota interactions and their implications for healthy living. *Nutrients* 5 234–252. 10.3390/nu5010234 23344252 PMC3571646

[B23] Jones R. B. Alderete T. L. Kim J. S. Millstein J. Gilliland F. D. Goran M. I. (2019). High intake of dietary fructose in overweight/obese teenagers associated with depletion of *Eubacterium* and *Streptococcus* in gut microbiome. *Gut Microbes* 10 712–719. 10.1080/19490976.2019.1592420 30991877 PMC6866686

[B24] Kalmokoff M. L. Teather R. M. (1997). Isolation and characterization of a bacteriocin (Butyrivibriocin AR10) from the ruminal anaerobe *Butyrivibrio fibrisolvens* AR10: evidence in support of the widespread occurrence of bacteriocin-like activity among ruminal isolates of *B. fibrisolvens*. *Appl. Environ. Microbiol.* 63:394. 10.1128/aem.63.2.394-402.1997 9023920 PMC168332

[B25] Karlsson C. L. Onnerfalt J. Xu J. Molin G. Ahrne S. Thorngren-Jerneck K. (2012). The microbiota of the gut in preschool children with normal and excessive body weight. *Obesity (Silver Spring)* 20 2257–2261. 10.1038/oby.2012.110 22546742

[B26] Kemp B. den Hartog L. A. Klok J. J. Zandstra T. (1991). The digestibility of nutrients, energy and nitrogen in the Meishan and Dutch Landrace pig. *J. Anim. Physiol. Ann.* 65 263–266. 10.1111/j.1439-0396.1991.tb00265.x

[B27] Koh A. De Vadder F. Kovatcheva-Datchary P. Backhed F. (2016). From dietary fiber to host physiology: short-chain fatty acids as key bacterial metabolites. *Cell* 165 1332–1345. 10.1016/j.cell.2016.05.041 27259147

[B28] Kuhad R. C. Gupta R. Singh A. (2011). Microbial cellulases and their industrial applications. *Enzyme Res.* 2011:280696. 10.4061/2011/280696 21912738 PMC3168787

[B29] Kyriazakis I. Emmans G. C. (1995). The voluntary feed intake of pigs given feeds based on wheat bran, dried citrus pulp and grass meal, in relation to measurements of feed bulk. *Br. J. Nutr.* 73 191–207. 10.1079/bjn19950023 7718540

[B30] Lan A. Bruneau A. Philippe C. Rochet V. Rouault A. Herve C. (2007). Survival and metabolic activity of selected strains of *Propionibacterium freudenreichii* in the gastrointestinal tract of human microbiota-associated rats. *Br. J. Nutr.* 97 714–724. 10.1017/s0007114507433001 17349084

[B31] Lindberg J. E. (2014). Fiber effects in nutrition and gut health in pigs. *J. Anim. Sci. Biotechnol.* 5:15. 10.1186/2049-1891-5-15 24580966 PMC3975931

[B32] Lu D. Tiezzi F. Schillebeeckx C. McNulty N. P. Schwab C. Shull C. (2018). Host contributes to longitudinal diversity of fecal microbiota in swine selected for lean growth. *Microbiome* 6:4. 10.1186/s40168-017-0384-1 29301569 PMC5755158

[B33] Navarro D. Bruininx E. de Jong L. Stein H. H. (2018). The contribution of digestible and metabolizable energy from high-fiber dietary ingredients is not affected by inclusion rate in mixed diets fed to growing pigs. *J. Anim. Sci.* 96 1860–1868. 10.1093/jas/sky090 29534181 PMC6140883

[B34] Navarro D. M. D. L. Bruininx E. M. A. M. de Jong L. Stein H. H. (2019). Effects of inclusion rate of high fiber dietary ingredients on apparent ileal, hindgut, and total tract digestibility of dry matter and nutrients in ingredients fed to growing pigs. *Anim. Feed Sci. Tech.* 248 1–9. 10.1016/j.anifeedsci.2018.12.001

[B35] Niu Q. Li P. Hao S. Kim S. W. Du T. Hua J. (2019). Characteristics of gut microbiota in sows and their relationship with apparent nutrient digestibility. *Int. J. Mol. Sci.* 20:870. 10.3390/ijms20040870 30781601 PMC6412398

[B36] Noblet J. Goff G. L. (2001). Effect of dietary fibre on the energy value of feeds for pigs. *Anim. Feed Sci. Tech.* 83 35–52. 10.1016/s0377-8401(01)00195-x

[B37] Owusu-Asiedu A. Patience J. F. Laarveld B. Van Kessel AG Simmins P. H. Zijlstra R. T. (2006). Effects of guar gum and cellulose on digesta passage rate, ileal microbial populations, energy and protein digestibility, and performance of grower pigs. *J. Anim. Sci.* 84 843–852. 10.2527/2006.844843x 16543561

[B38] Palm N. Dezoete M. Cullen T. Barry N. Stefanowski J. Hao L. (2014). Immunoglobulin a coating identifies colitogenic bacteria in inflammatory bowel disease. *Cell* 158 1000–1010. 10.1016/j.cell.2014.08.006 25171403 PMC4174347

[B39] Pu G. Huang R. H. Niu Q. Wang H. Fan L. J. Gao C. (2019). Effects of dietary defatted rice bran substitute corn levels on growth performance, intestinal development and apparent digestibility of nutrients of suhuai pigs. *Acta Veter. Zootech. Sin.* 50 758–770. 10.11843/j.issn.0366-6964.2019.04.009

[B40] Renteria-Flores J. A. Johnston L. J. Shurson G. C. Gallaher D. D. (2008). Effect of soluble and insoluble fiber on energy digestibility, nitrogen retention, and fiber digestibility of diets fed to gestating sows. *J. Anim. Sci.* 86 2568–2575. 10.2527/jas.2007-0375 18539846

[B41] Riva A. Kuzyk O. Forsberg E. Siuzdak G. Pfann C. Herbold C. (2019). A fiber-deprived diet disturbs the fine-scale spatial architecture of the murine colon microbiome. *Nat. Commun.* 10:4366. 10.1038/s41467-019-12413-0 31554820 PMC6761162

[B42] Shannon P. Markiel A. Ozier O. Baliga N. S. Wang J. T. Ramage D. (2003). Cytoscape: a software environment for integrated models of biomolecular interaction networks. *Genome Res.* 13 2498–2504. 10.1101/gr.1239303 14597658 PMC403769

[B43] Singh V. Yeoh B. S. Chassaing B. Xiao X. Saha P. Aguilera Olvera R. (2018). Dysregulated microbial fermentation of soluble fiber induces cholestatic liver cancer. *Cell* 175 679–694.e22. 10.1016/j.cell.2018.09.004 30340040 PMC6232850

[B44] Slavin J. (2013). Fiber and prebiotics: mechanisms and health benefits. *Nutrients* 5 1417–1435. 10.3390/nu5041417 23609775 PMC3705355

[B45] Tilg H. Kaser A. (2011). Gut microbiome, obesity, and metabolic dysfunction. *J. Clin. Invest.* 121 2126–2132. 10.1172/jci58109 21633181 PMC3104783

[B46] Tremaroli V. Backhed F. (2012). Functional interactions between the gut microbiota and host metabolism. *Nature* 489 242–249. 10.1038/nature11552 22972297

[B47] Valcheva R. Koleva P. Martínez I. Walter J. Gänzle M. G. Dieleman L. A. (2019). Inulin-type fructans improve active ulcerative colitis associated with microbiota changes and increased short-chain fatty acids levels. *Gut Microbes* 10 334–357. 10.1080/19490976.2018.1526583 30395776 PMC6546336

[B48] Varel V. H. Yen J. T. (1997). Microbial perspective on fiber utilization by swine. *J. Anim. Sci.* 75 2715–2722. 10.2527/1997.75102715x 9331875

[B49] Wang B. Li P. Zhou W. Gao C. Liu H. Li H. (2019a). Association of twelve candidate gene polymorphisms with the intramuscular fat content and average backfat thickness of Chinese Suhuai pigs. *Animals (Basel)* 9:858. 10.3390/ani9110858 31652864 PMC6912197

[B50] Wang J. Qin C. He T. Qiu K. Sun W. Zhang X. (2018). Alfalfa-containing diets alter luminal microbiota structure and short chain fatty acid sensing in the caecal mucosa of pigs. *J. Anim. Sci. Biotechnol.* 9:11. 10.1186/s40104-017-0216-y 29372054 PMC5769528

[B51] Wang X. Sun G. Feng T. Zhang J. Huang X. Wang T. (2019b). Sodium oligomannate therapeutically remodels gut microbiota and suppresses gut bacterial amino acids-shaped neuroinflammation to inhibit Alzheimer’s disease progression. *Cell Res.* 29 787–803. 10.1038/s41422-019-0216-x 31488882 PMC6796854

[B52] Wang X. Tsai T. Deng F. Wei X. Chai J. Knapp J. (2019c). Longitudinal investigation of the swine gut microbiome from birth to market reveals stage and growth performance associated bacteria. *Microbiome* 7:109. 10.1186/s40168-019-0721-7 31362781 PMC6664762

[B53] Wilfart A. Montagne L. Simmins H. Noblet J. Milgen J. (2007). Effect of fibre content in the diet on the mean retention time in different segments of the digestive tract in growing pigs. *Livest. Sci.* 109 27–29. 10.1016/j.livsci.2007.01.032

[B54] Xiao L. Estelle J. Kiilerich P. Ramayo-Caldas Y. Xia Z. Feng Q. (2016). A reference gene catalogue of the pig gut microbiome. *Nat. Microbiol.* 1:16161. 10.1038/nmicrobiol.2016.161 27643971

[B55] Zervas S. Zijlstra R. T. (2002a). Effects of dietary protein and fermentable fiber on nitrogen excretion patterns and plasma urea in grower pigs. *J. Anim. Sci.* 80 3247–3256. 12542166 10.2527/2002.80123247x

[B56] Zervas S. Zijlstra R. T. (2002b). Effects of dietary protein and oathull fiber on nitrogen excretion patterns and postprandial plasma urea profiles in grower pigs. *J. Anim. Sci.* 80 3238–3246. 10.2527/2002.80123238x 12542165

[B57] Zhang W. Li D. Liu L. Zang J. Duan Q. Yang W. (2013). The effects of dietary fiber level on nutrient digestibility in growing pigs. *J. Anim. Sci. Biotechnol.* 4:17. 10.1186/2049-1891-4-17 23587355 PMC3643821

[B58] Zhang Y. Q. Hao S. S. Gao S. Wu Y. Li Q. Li P. H. (2016). Effects of rice bran source high fibre diet on growth performance and intestine function of Suhuai pigs. *J. Nanjing Agric. Univ.* 39 807–813. 10.7685/jnau.201601011

[B59] Zhao J. Liu P. Wu Y. Guo P. Liu L. Ma N. (2018). Dietary fiber increases butyrate-producing bacteria and improves the growth performance of weaned piglets. *J. Agric. Food Chem.* 66 7995–8004. 10.1021/acs.jafc.8b02545 29986139

