# Supplementary material for: Adding Appropriate Fiber in Diet Increases Diversity and Metabolic Capacity of Distal Gut Microbiota Without Altering Fiber Digestibility and Growth Rate of Finishing Pig
Source: Front Microbiol. 2020 Apr 9;11:533. doi: 10.3389/fmicb.2020.00533 (PMC7160236; doi:10.3389/fmicb.2020.00533)
Supplement: Supplementary file 1 [file Data_Sheet_1.docx]

**Supplementary Table S1** The analyzed chemical composition of the corn and defatted rice bran (DM basis)

| Items | Corn | Defatted rice bran | *P*-value |
| --- | --- | --- | --- |
| CP, % | 10.62±0.12 | 18.54±0.18 | <0.000 |
| EE, % | 5.49±0.15 | 3.13±0.21 | <0.000 |
| CF, % | 2.42±0.19 | 10.88±0.08 | <0.000 |
| NDF, % | 11.12±0.25 | 30.09±0.47 | <0.000 |
| ADF, % | 3.24±0.10 | 11.39±0.08 | <0.000 |
| Cellulose, % | 2.93±0.11 | 6.84±0.45 | <0.000 |
| Hemicellulose, % | 7.82±0.21 | 18.44±0.46 | <0.000 |
| Lignin, % | 0.36±0.01 | 4.24±0.44 | <0.000 |
| IDF, % | 8.92±0.25 | 26.1±0.17 | <0.000 |
| SDF, % | 0.79±0.11 | 1.36±0.20 | 0.027 |
| TDF, % | 9.71±0.27 | 27.33±0.25 | <0.000 |

Note: All indicators are measured values.

**Supplementary Table S2** Comparison of linear growth rate between experimental groups and control

| Item | n | Slope | *P* value |
| --- | --- | --- | --- |
| Treatment Ⅰ | 7 | 0.6307 | 0.608 |
| Control group | 7 | 0.6062 |  |
| Treatment Ⅱ | 7 | 0.6766 | 0.172 |
| Control group | 7 | 0.6062 |  |
| Treatment Ⅲ | 7 | 0.6626 | 0.239 |
| Control group | 7 | 0.6062 |  |
| Treatment Ⅳ | 7 | 0.6675 | 0.242 |
| Control group | 7 | 0.6062 |  |

Note: *P* value is used to test the difference of slopes between two regression lines.

**Supplementary Table S3** Comparison of the microbial alpha diversity between treatment groups and control group in caecum and colon respectively.

| Sampling  location | Items | Groups | | | SEM | *P* value | |
| --- | --- | --- | --- | --- | --- | --- | --- |
|  |  | Control group | Treatment Ⅱ | Treatment Ⅳ |  | one-way ANOVA | linear |
| Caecum | Sobs | 771.29^Bb^ | 927.33^Aa^ | 781.83^ABb^ | 25.01 | 0.010 | 0.714 |
|  | Ace | 932.85^b^ | 1081.06^a^ | 940.16^b^ | 27.97 | 0.044 | 0.811 |
|  | Chao | 932.47^b^ | 1092.89^a^ | 949.70^b^ | 28.08 | 0.029 | 0.676 |
|  | Shannon | 4.30 | 4.68 | 4.26 | 0.08 | 0.054 | 0.879 |
|  | Simpson | 0.05 | 0.03 | 0.05 | 0.00 | 0.309 | 0.968 |
| Colon | Sobs | 888.43 | 959.57 | 877 | 22.50 | 0.280 | 0.860 |
|  | Ace | 1036.99 | 1127.68 | 1030.35 | 25.40 | 0.225 | 0.914 |
|  | Chao | 1045.61 | 1146.66 | 1047.38 | 27.42 | 0.236 | 0.979 |
|  | Shannon | 4.71 | 4.80 | 4.72 | 0.06 | 0.831 | 0.835 |
|  | Simpson | 0.03 | 0.03 | 0.03 | 0.00 | 0.975 | 0.981 |

Note: A adjustment for multiple comparisons was conducted using LSD. In the same row, values with no letter or the same letter superscripts mean no significant difference (*P* > 0.05), while values with different small letter superscripts mean significant difference (*P* < 0.05), and values with different small capital superscripts mean significant difference (*P* < 0.01).

**Supplementary Table S4** Comparison of the concentrations of SCFAs between treatment groups and control group in caecum and colon respectively (μmol/g digesta)

|  |  | Groups | | |  | | *P* value | | |
| --- | --- | --- | --- | --- | --- | --- | --- | --- | --- |
| Sampling location | Items | Control | Treatment II | Treatment IV | | SEM | | One-way ANOVA | Linear |
| Caecum | Acetate | 41.23 | 56.02 | 59.30 | | 3.69 | | 0.098 | 0.045 |
|  | Propionate | 19.03 | 24.25 | 30.21 | | 1.97 | | 0.06 | 0.019 |
|  | Isobutyrate | 0.45^Bb^ | 0.85^Aa^ | 0.93^Aa^ | | 0.07 | | 0.005 | 0.002 |
|  | Butyrate | 10.15 | 11.16 | 12.07 | | 0.71 | | 0.573 | 0.298 |
|  | Isovalerate | 0.54^Bb^ | 0.93^Aa^ | 0.59^Bb^ | | 0.05 | | 0.001 | 0.639 |
|  | Valerate | 1.71 | 1.49 | 1.52 | | 0.12 | | 0.719 | 0.518 |
|  | Total VFA | 73.11 | 94.71 | 104.61 | | 5.66 | | 0.059 | 0.021 |
| Colon | Acetate | 35.91^Bb^ | 50.59^ABab^ | 59.51^Aa^ | | 3.76 | | 0.027 | 0.009 |
|  | Propionate | 15.9 | 18.47 | 19.37 | | 0.99 | | 0.350 | 0.168 |
|  | Isobutyrate | 1.32 | 1.25 | 1.35 | | 0.10 | | 0.910 | 0.890 |
|  | Butyrate | 8.44 | 8.33 | 9.74 | | 0.51 | | 0.475 | 0.317 |
|  | Isovalerate | 1.75 | 1.51 | 1.39 | | 0.10 | | 0.363 | 0.167 |
|  | Valerate | 1.51^Aa^ | 1.09^Bb^ | 1.16^ABb^ | | 0.07 | | 0.020 | 0.026 |
|  | Total VFA | 64.84 | 81.24 | 94.47 | | 5.06 | | 0.051 | 0.016 |

Note: Adjustment for multiple comparisons was conducted using LSD. In the same row, values with no letter or the same letter superscripts mean no significant difference (*P* > 0.05), while values with different small letter superscripts mean significant difference (*P* < 0.05), and values with different small capital superscripts mean significant difference (*P* < 0.01).


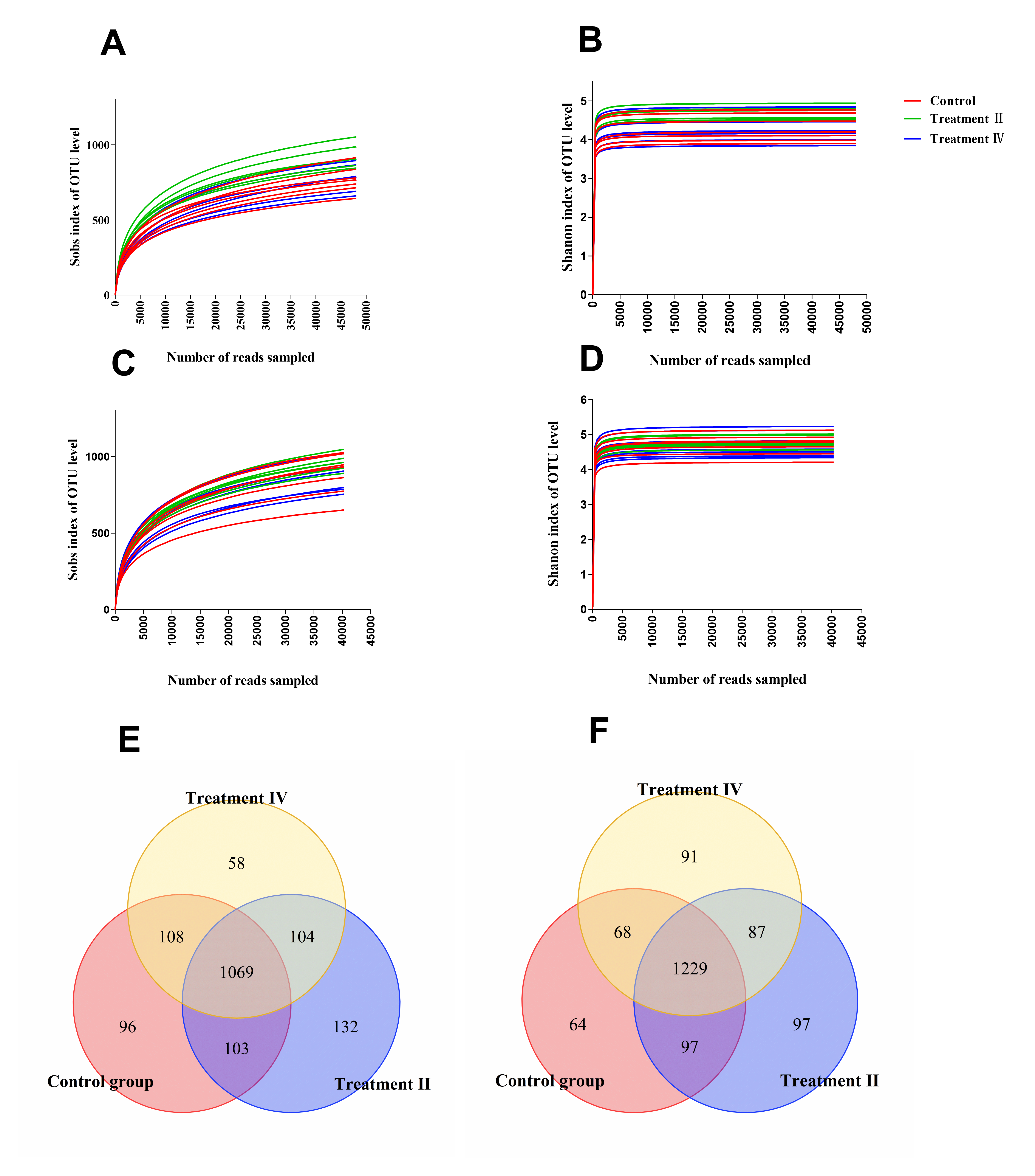


**Supplementary Figure S1.** The rarefaction curves in bacterial communities based on the Sobs index *A*) and the Shannon index *B*) in caecum and the rarefaction curves bacterial communities based on the Sobs index *C*) and the Shannon index *D*) in colon. Each group was shown by different line colors. Venn diagram of the caecal bacterial communities (*E*) and colonic bacterial communities (*F*) at OTU level.





**Supplementary Figure S2.** Relative change in the abundance of selected taxa in treatment groups relative to control. Only taxa with significant differences in population numbers are illustrated. *A* and *B*) Relative changes in the abundance of selected taxa in treatment Ⅱ, Ⅳ relative to control in caecum, respectively. *C* and *D*) Relative changes in the abundance of selected taxa in treatment Ⅱ, Ⅳ relative to control in colon, respectively. Flux was calculated by subtracting the relative abundance of taxa in control from the relative abundance in treatment groups. The green columns indicate more bacteria abundance in the treatment group than control group. The red columns indicate more bacteria abundance in the control group than treatment group.


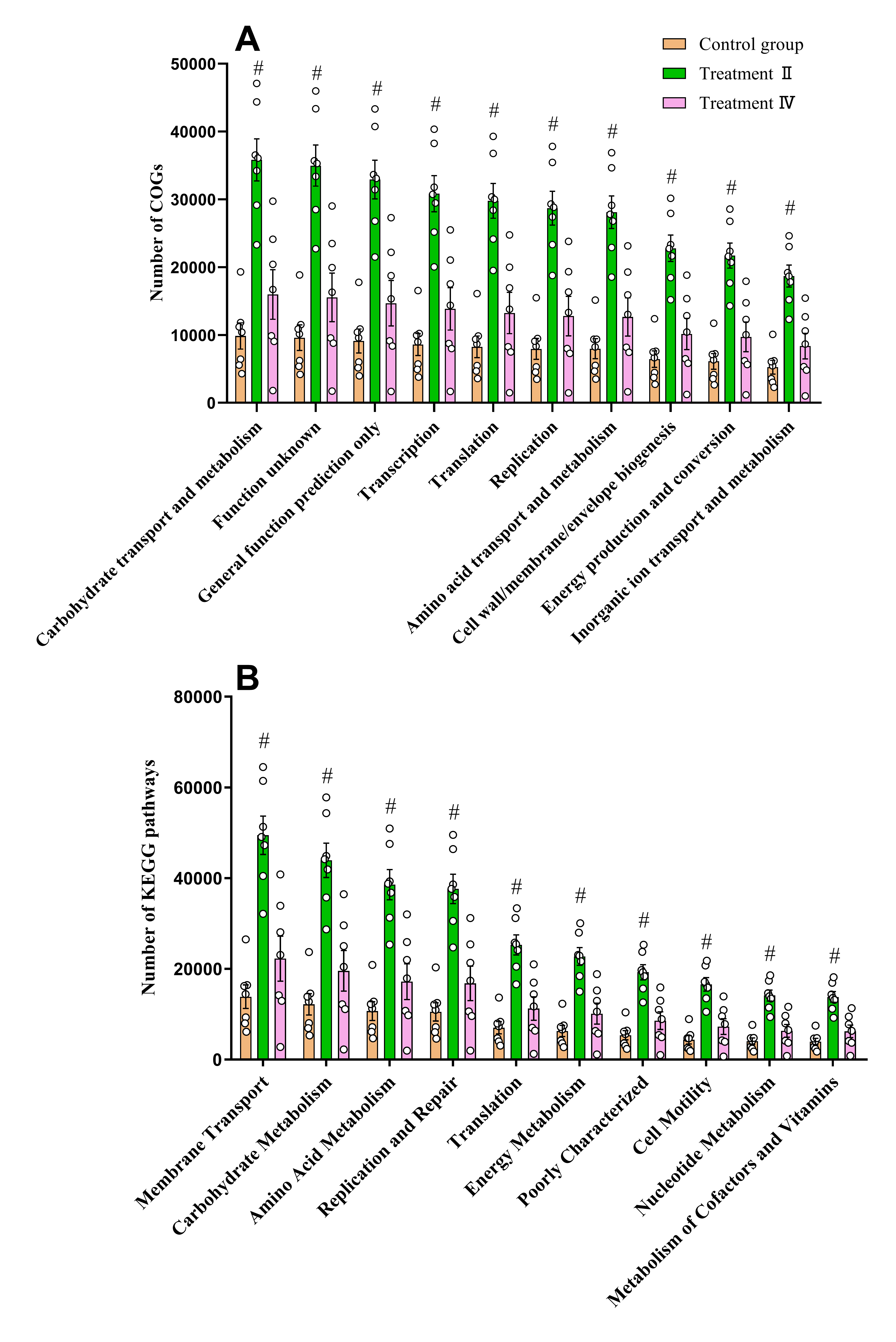


**Supplementary Figure S3.** Comparison of colonic microbial functions between treatment groups and control group.. *A*) Comparison of the top 10 microbial functions of COG among treatment Ⅱ, Ⅳ with control, respectively. *B*) Comparison of the top 10 microbial functions of KEGG among treatment Ⅱ, Ⅳ with control, respectively. ^#^*P* < 0.01 between treatment Ⅱ and control group.


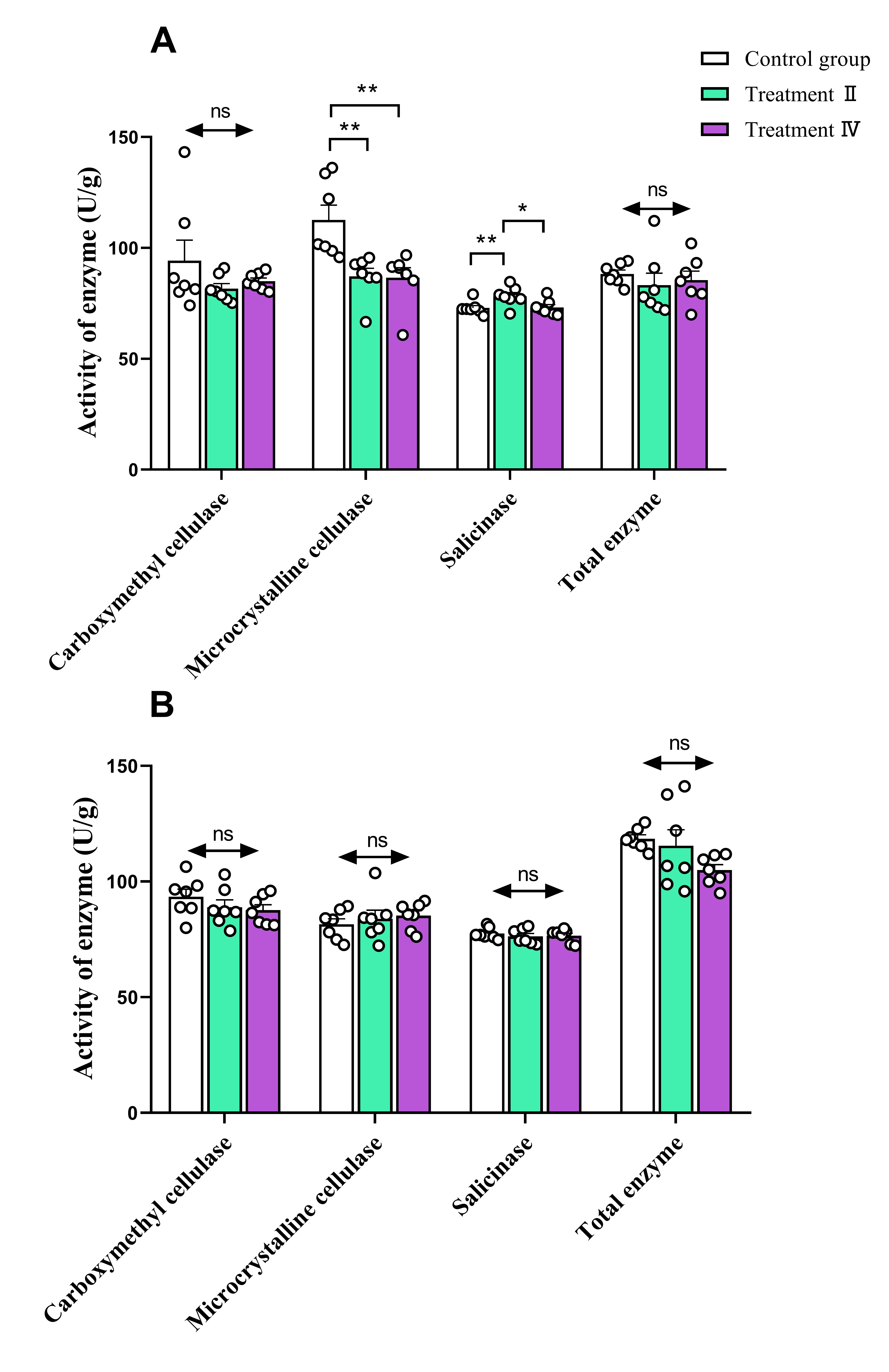


**Supplementary Figure S4** Comparisons of four fiber degrading enzymes among three groups in caecum (A) and colon (B). **P* < 0.05; ***P* < 0.01; ns, not significant.


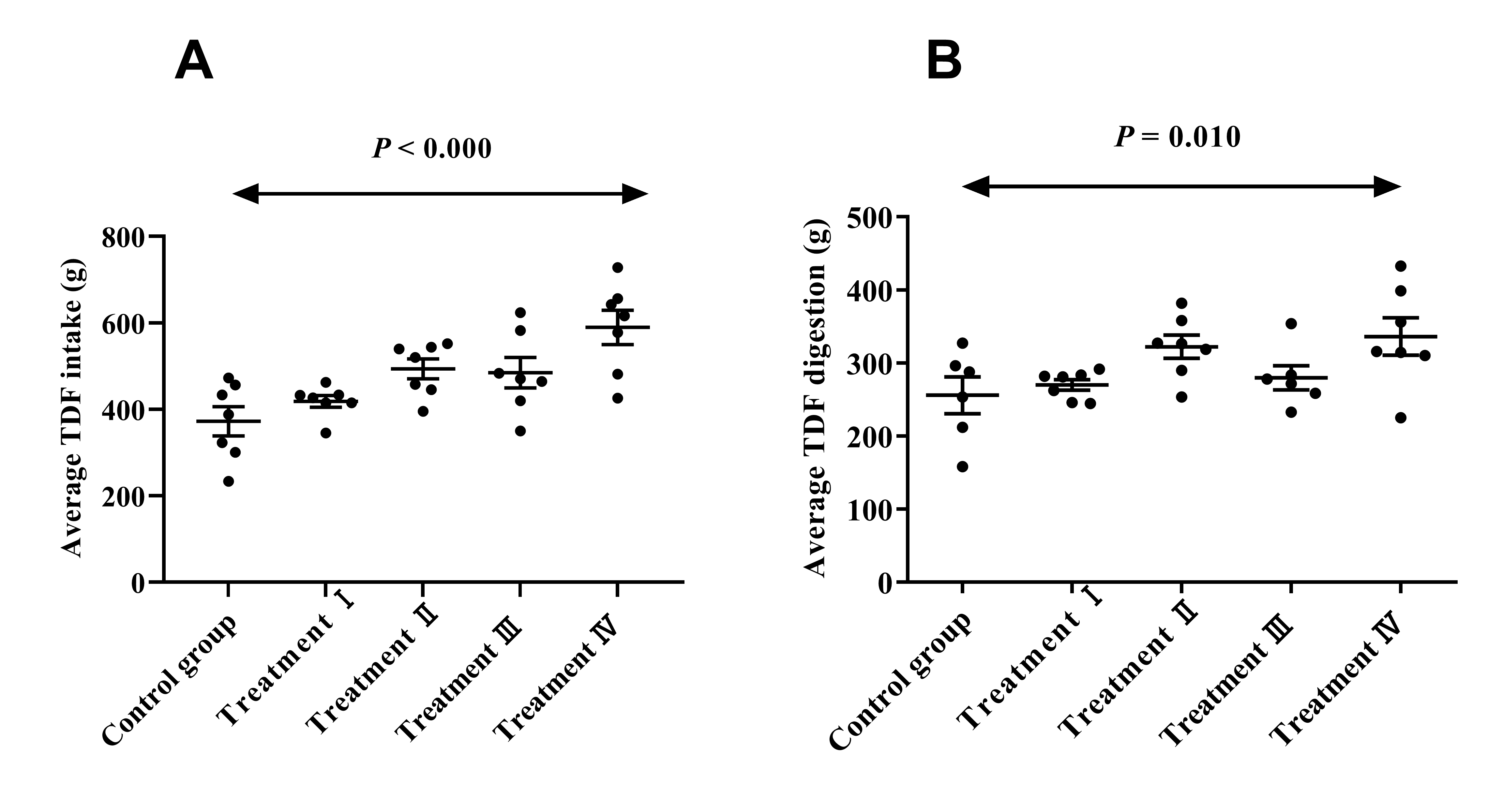


**Supplementary Figure S5.** *A*) The change of average total dietary fiber (TDF) intake among five groups. *B*) The change of average total dietary fiber digestion among five groups. One-way ANOVA was conducted.





**Supplementary Figure S6** Correlation network analysis among the concentrations of SCFAs and microbiota in colon. Networks display Spearman rank correlations among the relative abundance of microbiota and the concentrations of acetate (A), propionate (B), isobutyrate (C), butyrate (D), isovalerate (E), valerate (F) and total VFA (G). Square nodes represent the correlations with corrected values of 0.00 < *P* < 0.05, and round nodes represent the correlations with corrected values of 0.05 ≤ *P* < 0.10. Red lines represent positive correlation and green represent negative correlation.
